# Beta rhythm modulation by speech sounds: somatotopic mapping in somatosensory cortex

**DOI:** 10.1038/srep31182

**Published:** 2016-08-08

**Authors:** Eleonora Bartoli, Laura Maffongelli, Claudio Campus, Alessandro D’Ausilio

**Affiliations:** 1 Robotics, Brain and Cognitive Sciences (RBCS) Department, Italian Institute of Technology, Genova, Italy

## Abstract

During speech listening motor regions are somatotopically activated, resembling the activity that subtends actual speech production, suggesting that motor commands can be retrieved from sensory inputs. Crucially, the efficient motor control of the articulators relies on the accurate anticipation of the somatosensory reafference. Nevertheless, evidence about somatosensory activities elicited by auditory speech processing is sparse. The present work looked for specific interactions between auditory speech presentation and somatosensory cortical information processing. We used an auditory speech identification task with sounds having different place of articulation (bilabials and dentals). We tested whether coupling the auditory task with a peripheral electrical stimulation of the lips would affect the pattern of sensorimotor electroencephalographic rhythms. Peripheral electrical stimulation elicits a series of spectral perturbations of which the beta rebound reflects the return-to-baseline stage of somatosensory processing. We show a left-lateralized and selective reduction in the beta rebound following lip somatosensory stimulation when listening to speech sounds produced with the lips (i.e. bilabials). Thus, the somatosensory processing could not return to baseline due to the recruitment of the same neural resources by speech stimuli. Our results are a clear demonstration that heard speech sounds are somatotopically mapped onto somatosensory cortices, according to place of articulation.

Motor control theories advanced our understanding on the organization of movements by formalizing interactions between motor commands, sensory consequences of movements and action goals. In fact, the concept of internal models has been proposed to deal with the complexity of limb movement planning and execution, and to account for the required accuracy, in absence of very-short-latency feedback^1^. Unique to humans, speech production shares interesting analogies with limb motor control^2^, reason why internal model principles inspired research in speech motor control. Generally speaking, there are two quite different views regarding the primary variables controlled during speech production. On one side, the primary variables are articulatory gestures and the internal models make sure that effectors move along a specific desired trajectory in the vocal-tract space^3^. The alternative account suggests that the controlled variables are the specific patterns of auditory and somatosensory afferences^4^. Therefore, speech production goals are either specified in terms of articulatory or sensory parameters^5^.

However, the idea that during development the sensory consequences of the articulatory movement become tightly associated with the ongoing motor commands is common to both formulations. The association between motor commands and their sensory consequences builds forward models, whereas the reconstruction of motor commands needed to achieve a given sensory state or goal is captured by inverse models. Altering the sensory consequences of motor events can highlight internal models processes. Mismatching feedback with respect to sensory predictions enhances neural responses in auditory cortex regions^6^,^7^,^8^, non-overlapping with loci responsible for the attenuation response to self-generated speech^9^. Intriguingly, response attenuation effects can be modulated by the degree of mismatch, revealing a tolerance in speech motor control for sounds closer to the sensory target^10^. Compensatory changes occurring in feedback-altered speech are underpinned by the recruitment of a sensorimotor network^11^. Low-frequency brain oscillations might be responsible for signaling and orchestrating such compensatory mechanisms after prediction errors^12^, while a complex cross-frequency interplay subtends motor adaptation effects^13^, likely resembling adjustments in feedforward models^14^.

Paired forward-inverse models have been linked to the ability to learn and imitate^15^ as well as being a possible explanation for the mirror-like activity (i.e. similar neural activity between an individual performing a movement and an observer) which have been reported over frontal and parietal regions in the primate brain^16^. Crucially, there is relevant evidence that the exposure to speech sounds activates, in the listener, the missing information relative to that acoustic event. Namely, the articulatory pattern and the somatosensory feedback associated to that distal event.

Behavioral studies demonstrated that specific articulatory commands are involuntarily activated during speech perception. In one study, participants produced target syllables while listening to the same syllables or to incongruent ones. Tongue position during production, measured with electropalatography, revealed a clear contamination from the incongruent auditory distractor^17^. Moreover, a direct neurophysiological evidence substantiating that speech listening evokes automatic and congruent motor activities has been obtained by assessing the excitability of the corticobulbar tract, via Transcranial Magnetic Stimulation (TMS). Several studies have shown that speech listening evokes motor activities in a somatotopic manner^18^,^19^,^20^,^21^,^22^,^23^,^24^.

Additionally, behavioral evidence suggests that specific somatosensory processes are automatically activated during speech perception. In one study^25^, participants listened to speech sounds while discriminating facial skin deformations that would normally be associated to the production of those sounds. Performance in the somatosensory discrimination task was altered by speech sounds when auditory and somatosensory stimulations mismatched. In agreement with this result, it has been shown that somatosensory stimulations, associated with specific articulatory movements, are able to affect the perceptual processing of speech sounds^26^,^27^. Neurophysiological evidence corroborating the idea that speech perception evokes automatic and congruent somatosensory activities has been obtained via Somatosensory Evoked Potentials (SEPs) recorded through electroencephalography (EEG). Tactile stimulation to the lower lip coupled to speech viewing was shown to enhance early left components of SEPs, although no cross-talk was detected with speech listening alone^28^. More recently, tactile facial stimulation (skin stretch) during continuous speech listening showed a left-lateralized enhancement of the first negative peak of the SEPs^29^. Intriguingly, the amplitude of the SEPs varied as a function of the relative temporal asynchrony between the two sensory modalities^30^. These latter investigations did not show any somatotopic specificity of these somatosensory activities elicited by speech presentation.

The investigation of sensorimotor processing with EEG recordings has also exploited peripheral somatosensory stimulation, which has been shown to elicit a well known pattern of time-locked spectral perturbations. The pattern is characterized by a first Event-Related Desynchronization (ERDs), which is believed to represent the engagement of sensorimotor cortex^31^, occurring in specific frequency bands such as the beta (13–30 Hz) and mu rhythms (8–12 Hz-following the naming convention suggested to differentiate mu and alpha rhythms, being recorded over perirolandic versus posterior regions^32^,^33^). The ERD is later followed by an Event-related synchronization phase (ERS), which represents the return-to-baseline stage following sensorimotor engagement^34^, called for such reason also rebound. The ERS phase bears relevant properties that are of great interest in the investigation of sensorimotor brain dynamics during speech perception. In fact, the beta rebound is diminished or even abolished by various tasks involving the activity of the sensorimotor system, such as actual movement execution and even motor imagery^35^. Due to the fact that the ERD/ERS pattern is evoked by the specific stimulation of one localized bodily area, this effect can be used to investigate the functional somatotopy of the sensorimotor strip^36^.

In the current study, we intend to measure the pattern of ERD/ERS triggered by peripheral stimulation of a speech articulator during a speech identification task. To our knowledge, this approach has never been used to investigate the sensorimotor nature of speech auditory encoding. Our aim is to investigate the functional match between speech auditory and sensorimotor processing, by exploiting the known beta rebound effect and its dampening. To this end, we delivered peripheral lower lip electrical stimulation while subjects were listening to speech sounds, differing on place of articulation. Place of articulation could either match with the electrical stimulation (bilabials syllables) or not (dental syllables).

We predict that the somatotopic matching between the place of articulation of the speech sounds and peripheral electrical stimulation will induce a significantly larger attenuation of the beta rebound power in perirolandic regions. The concurrent activation of lip somatosensory cortex by bilabial sounds and peripheral stimulation would be reflected by the attenuation of the return-to-baseline stage (ERS) following sensorimotor engagement. This effect would demonstrate that auditory processing of speech recruits the same neural populations activated by a somatosensory stimulation, in a somatotopically specific manner. In contrast, an absence of syllable-specific modulations would suggest that patterns of sensorimotor rhythms cannot differentiate the place of articulation of speech sounds. This result would support the claim that listening to speech does not directly engage specific sensorimotor processing *per se* but rather these activities could be a delayed byproduct of associative processes located elsewhere.

## Results

**Figure 1:**
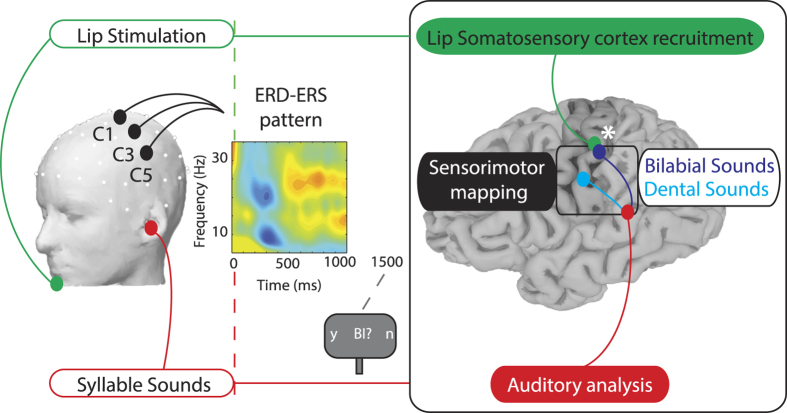
Experimental Design and Neurophysiological hypothesis. On the left side a schematic of the experimental design: Lip Stimulation to the lower lip (which could be either present or absent) is shown in green and the Syllable Sound presentation (which could either be bilabial or dental syllables) is depicted in red. Electrodes within the left ROI (C1, C3, C5) are represented on the head (drawn by using EEGLAB functions^72^, version 13.4.4b, site URL http://sccn.ucsd.edu/eeglab/). In the center of the figure a Time-Frequency map of the ERSP, displaying the ERD and ERS pattern. Electrical stimulation to the lips and vowel’s onset are temporally aligned (red and green dashed lines at time = 0 ms). After 1500 ms a question regarding the heard sound appeared on the screen, requiring participants to respond using horizontal eye movements. On the right a schematic of the Neurophysiological Hypothesis: if speech is represented in a sensorimotor code (black box), each sound (after auditory analysis, in red) will be mapped onto distinct portions of the somatosensory cortex (post-central gyrus) depending of the specific place of articulation of the syllable (cyan or dark blue). Peripheral stimulation of the lips (in green) will match with one of the two somatosensory representations (bilabial sounds, dark blue). The white asterisk depicts the interaction between the processing of lip-related sounds with the lip-stimulation, due to the recruitment of overlapping neural resources (brain surface reconstructed from the MRI of one author using Freesurfer image analysis suite^75^, version 5.3.0, site URL http://freesurfer.net).

Participants were required to attend the auditory syllables (independent variable with two levels: dental/bilabial syllables) while EEG was recorded continuously. Lower lip stimulation, delivered in half of the trials, occurred aligned to vowel onset (independent variable with two levels: stimulation present/absent). The correct encoding of the sound was monitored by asking an identification question at the end of each trial (Fig. 1). Trials related to an incorrect response (13.1%) were discarded from further analysis.

EEG traces were used to calculate event-related spectral perturbations (ERSPs) by means of a wavelet decomposition of the voltage values recorded over a left central region of interest (left ROI: electrodes C1, C3 and C5) and over the corresponding right electrodes as a control (right ROI: C2, C4 and C6). The ROIs included central electrode locations classically employed to investigate ERD/ERS patterns^31^, being located above perirolandic regions. Given our hypothesis, the investigation of other speech-related regions was beyond the scope of the present study. To explore the effect of sound processing and of peripheral stimulation, we contrasted the ERSPs related to the two syllables separately for trials with and without stimulation.

**Figure 2:**
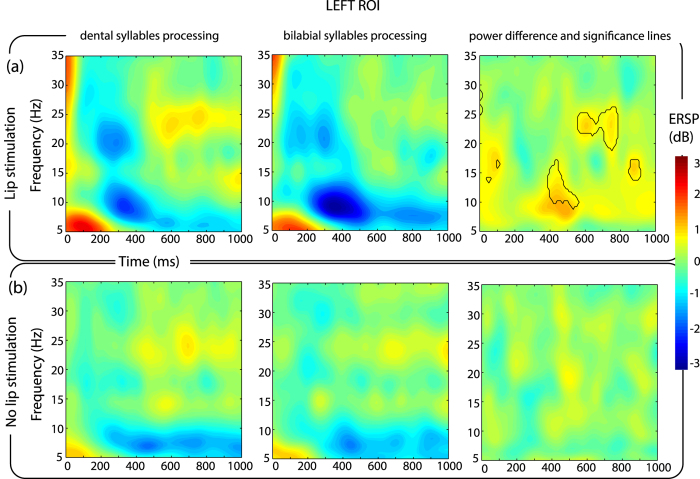
ERD/ERS pattern over left electrodes. Time-Frequency maps of ERSP in the left ROI for each combination of the experimental conditions. The x axis represents time in milliseconds (Time = 0 ms corresponds to the vowel onset and when present, to electrical stimulation), the y axis represents frequency in Hz. The color represents the magnitude of the ERSP in dB. The upper row (panel a) shows the trials related to lip stimulation. The lower row (panel b) trials without lip stimulation. For each panel, the left column shows the ERSPs while listening to dental syllables (/di/), the central column for the bilabial syllables (/bi/). The third column on the right represents the difference between/di/ and /bi/. On the right column, the black-lines show the time-frequency points related to a significant difference between the processing of the two syllables (p < 0.01, FDR corrected).

With respect to stimulation trials, mean ERSPs in the left ROI revealed the presence of an ERD/ERS pattern in the beta and mu frequency ranges. The ERD occurred in the beta frequency range (in particular, in the 16–25 Hz band) and in the mu frequency range (8–12 Hz) between 200 and 400 ms after electrical stimulation in both listening conditions (/bi/and/di/syllables). An ERS covering almost the whole beta range (13–30 Hz) and mu range (8–12 Hz) followed the ERD at around 500 ms and lasting about 300 ms. The comparison of ERSPs between dental and bilabial syllables processing revealed the time-frequency points in which a statistical difference (p < 0.01 with FDR correction) occurred between the syllables. These difference were found in ERS phase, in particular in the mu range (8–12 Hz) between 400–600 ms and in the upper beta range (20–26 Hz) between 600–800 ms (Fig. 2a).

**Figure 3:**
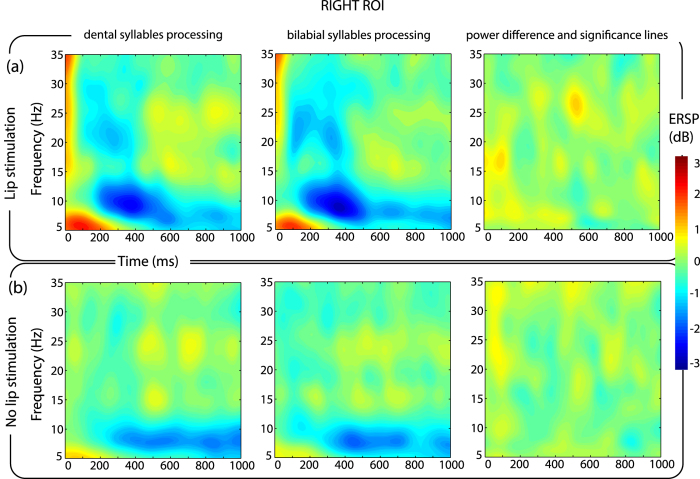
ERD/ERS pattern over right electrodes. Time-Frequency maps of ERSP in the right ROI for each combination of the experimental conditions. By comparison with the left ROI (Fig. 2), it is possible to notice the absence of any significant difference related to the processing of the two sounds for both stimulation and no stimulation trials.

Mean ERSPs in the right ROI shows an ERD in the beta and mu frequency range, followed by a low power ERS in the beta frequencies. No differences were found to be statistically significant between the two syllables in the right ROI (Fig. 3a).

Considering trials without stimulation, no difference could be detected between the processing of the two syllables, both over the left (Fig. 2b) and right ROIs defined (Fig. 3b).

The ERSP values from the left ROI were extracted for the two frequency bands of interest and averaged across two time windows capturing the ERD and the ERS events (200–400 ms for ERD time window; 400–600 ms for mu ERS, 600–800 ms for beta ERS time window; time windows based on the previous analysis). The ERSP values were analyzed using a within-subject ANOVA by means of a 2 (dental/bilabial auditory syllables) × 2 (stimulation present/absent) × 2 (ERD/ERS time window) full-factorial design for each frequency (mu and beta) separately.

### Beta-band ERD/ERS pattern

The ANOVA model showed that beta ERSP was significantly affected by the stimulation, and that it also differed across the ERD and ERS time windows (main effect of stimulation F(1, 11) = 10.04, p = 0.0089; main effect of time window, F(1, 11) = 25.71, p = 0.00036), by exhibiting a desynchonization effect in the ERD time window (mean ERSP −0.5238 ± 0.2498 dB), and a synchronization effect in the ERS time window (0.4482 ± 0.2595 dB) consistent with the time-course of the effect of somatosensory stimulation. An interaction between the stimulation and the time window (F(1, 11) = 7.794, p = 0.0175) was also found, due to the strong desynchronization induced by the stimulation in the ERD time window only (mean ERSP with stimulation −0.9703 ± 0.3003 dB, without stimulation −0.0772 ± 0.2595 dB). An interaction between the heard syllable and the time window was also present (syllable and time window interaction, F(1, 11) = 5.01, p = 0.0468), driven by the difference in mean ERSP between the two syllables in the ERS time window (mean ERSP for bilabial syllables 0.2483 ± 0.2835 dB, dental 0.6481 ± 0.2479 dB) which was not present in the ERD time window (ERSP bilabial −0.5482 ± 0.2913 dB, dental −0.4993 ± 0.2456 dB).

**Figure 4:**
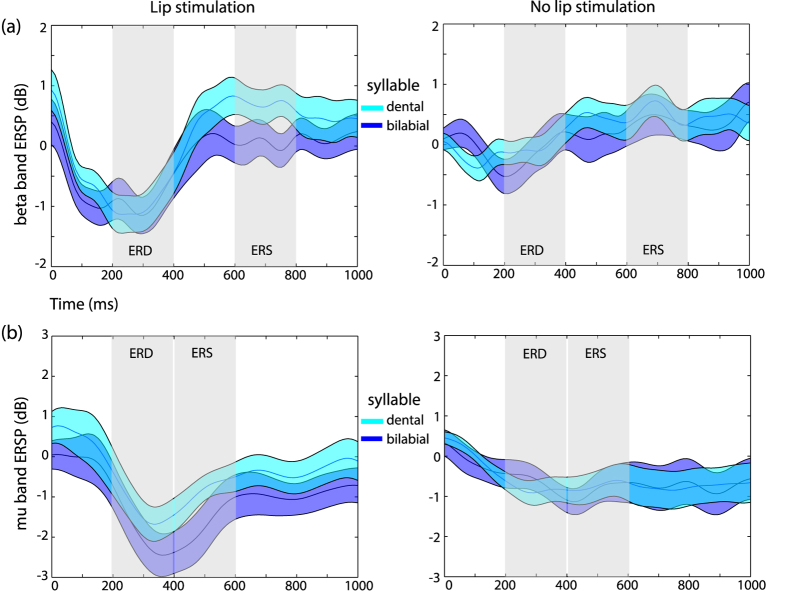
Beta and mu rhythms modulations by speech sounds. Time course of ERSP in the beta (panel a) and mu (panel b). The left columns show the result for the stimulation trials, the right for no stimulation trials. Shaded areas indicate the time windows of the ERD and ERS selected for the analysis, based on the exploration of the spectrograms. ERSP related to the processing of dental syllables are depicted in cyan, whereas for the bilabial syllables the traces are in dark blue. Shading around the traces represents two standard errors from the mean.

A three-way interaction was also present between syllable, stimulation and time (F(1, 11) = 5.413, p = 0.0401), showing not only that the difference between the two syllables was present in the ERS window, but also that it was driven by the presence of stimulation (ERSP bilabial 0.0438 ± 0.2909 dB, dental 0.7362 ± 0.2767 dB) and negligible when stimulation was absent (ERSP bilabial 0.4528, ±0.3201 dB, dental 0.5599 ± 0.2489 dB). No other main effects or interactions were found to be statistically significant. To confirm that the interaction between the experimental factors resembled our expected result, post-hoc comparisons testing the difference between the two syllables in the ERS time window were performed in both stimulation and no stimulation trials (two-tailed paired t-test, true alpha level with Bonferroni correction for two comparisons α = 0.025). The results confirmed a significant difference due to a reduced beta ERS in stimulation trials during the listening of bilabial syllables with respect to dental ones (t(11) = 4.9894, p = 0.000041). This difference was not significant during no-stimulation trials (t(11) = 0.5612, p = 0.5859) (Fig. 4a).

### Mu-band ERD/ERS pattern

Regarding the mu ERSP, a modulation depending on the presence of stimulation was found, due to the strong desynchronization induced by stimulation (mean ERSP for stimulation trials −1.5525 ± 0.4259 dB vs ERSP for trials without stimulation −0.7066 ± 0.3313 dB, main effect of stimulation F(1, 11) = 8.339, p = 0.0148). The difference between stimulation and no stimulation trials was also dependent on the time window considered, being more pronounced in the ERD time window (ERSP in ERD window for trials with stimulation −1.8481 ± 0.4525 dB, for trials without stimulation −0.7386 ± 0.2851 dB; in ERS window for stimulation −1.2568 ± 0.4648 dB, for no stimulation −0.06747 ± 0.4139 dB; stimulation and time window interaction F(1, 11) = 5.131, p = 0.0447). No other main effect or interaction reached significance, indicating that power changes in the mu frequency band were not affected by the auditory syllables or by their interaction with other variables (Fig. 4b).

## Discussion

The present study aimed at identifying the signature of the specific functional match between speech auditory information processing and sensorimotor mapping of peripheral somatosensory information. We exploited the somatotopic-specific ERD/ERS pattern^36^, which reliably follows self paced movements^37^, action observation^38^, motor imagery^35^ and, most importantly, the peripheral stimulation of nerves^39^ to target lip-somatosensory cortex activity. Here, by applying lip electrical stimulation, we tested if speech sounds are mapped onto sensorimotor neural circuits of speech. Critically, if speech processing uses specific sensorimotor neural resources, we should expect an interaction between speech sounds and the ERD/ERS pattern evoked by peripheral stimulation of the lips.

A clear ERD/ERS pattern is evoked by the electrical stimulation of the lower lips. The ERD in the beta and mu frequency bands occurred between 200 and 400 ms from stimulation and it was followed by an ERS, more pronounced in the beta band, starting at around 500 ms and lasting 300 ms. Importantly, the concurrent processing of speech sounds did affect the ERD/ERS pattern according to the match between the place of articulation of the syllable and the site of peripheral somatosensory stimulation. More specifically, we report a decreased ERS in the beta-band power when lip-stimulation was coupled with the processing of bilabial sounds with respect to dental sounds.

First of all, this result shows the interaction between sensorimotor encoding of peripheral somatosensory stimuli and auditory speech processing. This is in agreement with studies investigating brain oscillatory patterns during human actions observation^40^,^41^ and in response to median nerve stimulation and actions observation^42^,^43^,^44^. In these latter studies it was suggested that the mirror neurons system, known to directly facilitate the motor output during observation of actions^45^, may also modulate somatosensory activity in post-central areas^43^. Here we extend these results to the speech domain, by demonstrating that a specific pattern of beta ERD/ERS following lip-stimulation can be modulated by speech perception.

Secondly, in the present experiment the interaction between sensorimotor and auditory processing is specific to the left hemisphere. In fact, electrical stimulation was applied to the midline of the inferior lips, but effects were limited to the left hemisphere. ERD/ERS patterns and modulation thereof, are naturally lateralized to the hemisphere contralateral to the peripheral stimulation site. We argue that the linguistic content of the auditory stimulation might have biased the modulatory effects towards the left side, in agreement with the well-known left hemispheric dominance for speech and language processing. This pattern also matches the left lateralization shown in previous studies investigating SEPs modulations during speech processing^28^,^29^,^30^.

Thirdly, modulation of ERD/ERS during speech processing is specific to the beta band, whereas the mu band is not differentiating between syllables. Previous EEG studies on sensorimotor rhythms associated to speech discrimination in the absence of somatosensory stimulation, revealed significant suppression in the beta frequency range prior to, during, and following syllable discrimination trials^46^,^47^, which has been ascribed to a predictive-anticipatory effect. At the same time, speech discrimination was also characterized by early mu ERS prior to and during stimulus presentation, followed by mu ERD after stimulus offset^47^, interpreted as a response-selection effect. However, in our experiment, differently from these investigations, we did not search for frequency-modulations induced by auditory speech processing alone. We instead exploited peripheral lip stimulation to target neural activities that are specific to the processing of afferent lips information. Analogously, during action observation, a dissociation between beta and mu ERD/ERS patterns has been previously reported, similar to the one in the present study, with the beta rebound following peripheral stimulation being reduced during movement observation, whereas the mu band showed almost no modulation^43^. Other frequency bands, not directly addressed in the present study, are known to play a role in speech processing. For example, gamma band power is believed to represent local cortical processing and has been implied in predictive coding for speech^9^ as well as for speech motor adaptation effects, through an interplay with both theta and beta rhythms^13^. The present experiment was not designed to disentangle the roles played by these frequency bands and future studies will be needed to assess their contribution in the somatosensory mapping of speech sounds.

Thus, in order to put these results in context, it is important to consider the functional meaning of the sensorimotor rhythms analyzed. In general, the increase in beta synchronization corresponds to a reduced corticospinal excitability^48^. Indeed, it has been proposed that the beta rhythms convey motor information (efferent copies) to suppress self-generated sensory stimulations, freeing up resources to respond to external sensory stimuli^49^. In fact, beta rhythms interact with low frequency rhythms to anticipate sensory events by boosting the gain of neural responses to sensory signals^50^. The beta rebound elicited by peripheral electrical stimulations originates mainly in the contralateral primary somatosensory cortex^51^. It is considered to represent resetting and re-synchronization of the cortical oscillations^35^ following the desynchronization induced by somatosensory information processing. Therefore, more than an exclusively motor function, beta rhythms seem to play a critical role in connecting motor and sensory information processing. In fact, it is important to note that the post movement beta rebound is sensitive to the type and amount of afferent input^52^. Therefore, during action observation and, as suggested here in speech perception, the somatosensory stimulation highlights mechanisms of storage and encoding of somatosensory information, which are tightly associated with other sources of sensory information related to the perceived movement, such as its acoustic representation. The association between sensory consequences of motor commands allows the brain to build sensorimotor maps, which are at the foundation of motor control principles. To accomplish successful speech production, internal models rely on the efference copy to monitor the achievement of the sensory goal^10^. This has been accurately characterized using real-time speech distortions during speech production^6^,^9^,^11^. Internal models have also been evidenced during speech processing, as internal simulation and speech imagery are able to affect neural responses to speech perception^53^,^54^, due to recurrent estimations of auditory and somatosensory predictions^55^.

Finally, our results demonstrate that sensorimotor and auditory speech processing are matched according to their articulatory somatotopy. The beta rebound dissociates according to somatotopic representation during action execution^36^,^56^ or imagery^35^. Here we show that the beta ERS following peripheral stimulation is modulated by the different place of articulation of the auditory stimuli. This result clearly shows that auditory presentation of speech stimuli triggers neurally specific sensorimotor processes in the listener. This level of somatotopic specificity, was achieved without resorting to any source localization technique, and based solely on the characteristics of the stimuli. In fact, such spatial accuracy was achieved by presenting peripheral stimulations localized to the somatosensory field of one effector, temporally aligned to speech sounds produced with same or a different effector. It has been shown that the ERD/ERS patterns are somatotopically mapped according to the stimulated body regions^36^, which in the present experiment is the lip somatosensory cortex. The ERD/ERS pattern was modulated specifically by bilabial sounds only, which, as sustained by internal models of speech, are associated to lip movements and sensory afferences. This specific interaction with the auditory stimuli allows us to infer the cortical overlap by means of a functional criterion. In our opinion, investigations based on a functional rather than an algorithmic segregation of the neural sources are to be preferred, especially when investigating largely overlapping neural representations such as the lips and the tongue sensorimotor areas. To our knowledge, this is the first demonstration of a somatosensory somatotopy for speech perception.

We believe this is a critical new result given the fact that, within speech motor control, a fundamental role is played by the specific somatosensory reafference^57^,^58^. More importantly, we also know that somatosensory inputs affect the neural processing of speech sounds^26^,^27^, and speech sounds bias the discrimination of somatosensory input^25^. Critically, in order to observe these cross-modal interference phenomena, it is fundamental that stimulations in both modalities do match on a common motor ground. Somatosensory stimulation has to match the afferent information generated during the production of that very sound, in order to bias perception. Indeed, tactile information related to mouth movements, although experienced on the hands, affects speech perception when mismatching^59^. This results is also supported by the demonstration that early auditory evoked responses are attenuated and speeded up during audio-haptic speech perception when compared to auditory alone^60^. Altogether, these results support the hypothesis that our brain stores the diverse sources of information available with respect to the same speech event, to form distributed maps of correlated information across different neural systems (auditory, motor, somatosensory).

These maps emerge during development, through the pairing of speech production, auditory effects and the somatosensory afferences arising from articulation. The repeated and reliable association of these different source of information forms the internal models for speech. Strikingly, sensorimotor information from the articulators influences speech perception also in prelinguistic infants, without specific listening experience^61^. This result suggests that even during the initial stages of development, oral–motor non linguistic spontaneous movements enable the emergence of these multimodal maps. Later in development, the linguistic context of the child, fine-tune this ability. In fact, 7-months old infants activate auditory and motor brain areas in response to both native and non-native speech. Infants around their first year instead show greater auditory activities for native speech and greater motor activities for non native speech, matching the pattern usually found in adults^62^. This neural pattern follows the development of a behavioral preference for native versus non native speech sounds, along the first year of life^63^. These results clearly point to the key functional role potentially played by motor representations in speech perception^64^.

In fact, within the theoretical framework of internal models^15^, sensory information feeds an inverse model to retrieve the underlying motor commands, that in turn may generate sensory hypothesis to be compared with incoming new sensory features. These top-down processes might enhance perception by constraining the sensory search space^65^. In everyday life, this kind of predictive modeling can be used to cope with the increasing task demands related to foreign speech perception^66^, fast speech segmentation^67^, dealing with a noisy environment^68^,^69^, inter-speaker variability^70^, or compensating for low hearing acuity^24^. The present study adds the first neural demonstration that the somatosensory component may play a significant role in the predictive coding of speech perception. Future research is needed to understand how exactly the somatosensory predictive component interfaces with the motoric one during speech processing.

## Methods

### Participants

12 healthy right-handed healthy participants took part in the study (3 males, mean age: 28, SD: 5 years). All subjects had normal hearing and normal or corrected to normal vision and they gave informed consent to the experimental procedures, which were approved by the local ethics committee (Azienda Sanitaria Locale, Local Health Unit, Genoa) and were in accordance with the Declaration of Helsinki. Subjects were paid for their participation in the study.

### Stimuli

Auditory stimuli were consonant-vowel (CV) syllables, which were a subset of a stimuli described elsewhere^70^. The syllables were /bi/(bilabial) and /di/(dental), uttered by 6 different speakers, 3 males and 3 females. Production of bilabial syllables requires the distinctive movement of lips, whereas dental syllables require mainly a tongue movement against the upper teeth^71^. This set of stimuli was created to ensure a large variation in acoustic characteristics of the speech sounds, thus resembling an ecological scenario.

Somatosensory stimuli were induced by an electrical stimulation on the lower lip (DS7AH, Digitimer, Hertfordshire, UK). The cathode electrode was placed on the midline of the lower lip, the anode 1.5 cm on the right. The electrical stimulus lasted 200 microseconds with an average intensity of 30 mA. The intensity level was adjusted on individual basis (ranging from 25 to 35 mA) in order to be clearly identified without causing discomfort. Electrical stimulation, when present, was delivered in correspondence to the syllable vowel onset (see Fig. 1).

### Task, procedure and design

The subject was seated at approximately 70 cm distance from a computer screen. Subjects were asked to carefully listen the syllable presented through the earphones, while fixating a cross presented in the middle of the screen, and to identify the syllable by responding to a question. The response frame appeared 1.5 sec after the vowel’s onset and contained an explicit question regarding the syllable identity (see Fig. 1). The question was pseudorandomized in order to require half of the times a positive or a negative response. Subjects were requested to answer via saccadic eye movements towards the right or left sides of the screen. Eye-movements responses were used to avoid confounds related to effects of hand motor preparation on sensorimotor rhythms. The positioning of the positive and negative responses on the left or right sides was counterbalanced across subjects. This trial design impeded any anticipation of saccade location or response type. After a variable amount of time, between 7 and 8 seconds, a new trial started.

The procedure consisted in the pseudo-randomized presentation of the two types (bilabial/dental) of auditory syllables. Each of the 12 audio files was repeated 8 times, leading to a total of 48 trials for dental syllables and 48 trials for bilabial syllables. The speech sounds entered in a 2 (sound: bilabial/dental) × 2 (lip stimulation: present/absent) factorial design, leading to 192 trials (96 trials with stimulation, 96 without stimulation) in the experimental block (lasting around 1 hour).

### EEG data collection and preprocessing

A 64-channel EEG-System with an actiCap system^®^ (Brain Products, München, Germany) was used for data acquisition. Triggers were sent, through the parallel port, to the EEG system (Brain Amp MR Plus and ActiCap, Brain Products, München, Germany) and to the electrical stimulator device. All events were controlled by a Matlab (Mathworks, Inc.) script using Psychtoolbox functions.

EEG activity was recorded in the international 10–20 system with a physical reference electrode on FCz. Impedance was kept below five kohm (kΩ) during the whole acquisition. Two electrodes, placed at the outer part of each eye (outer canthi), recorded horizontal eye movements (EOG). The EEG signal was sampled at 500 Hz and data was acquired in continuous mode using Brain Vision Recorder (Brain Products, München, Germany). EOG data was exported separately for further processing. EEG data was band-passed (0.5 to 50 Hz) and then subsampled (250 Hz) with Brain Vision Analyzer 2.0 (Brain Products, München, Germany) and then exported for further analysis using EEGLAB toolbox^72^. The electrical stimulation produced a high-voltage artifact in the EEG recordings, lasting around 4 milliseconds. Artifacts related to the electrical stimulator artifact, eye movements, EMG activity and line current were removed through visual inspection by Independent Component Analysis (ICA) considering time, topographic and spectral distribution of the components. As an observation, the ICA algorithm was not able to isolate completely the electrical artifact for all the datasets. For our analysis, this was not problematic given the interest in later time windows, but it should be kept into consideration in studies interested in earlier components. Raw recordings were segmented 1.5 seconds before and 2.5 seconds after the marker signaling the vowel’s onset of the syllable. Segmented recordings were re-referenced using the average of all connected electrodes, baseline corrected (using the silent prestimulus time window from −1000 to −150 ms with respect to vowel onset).

## Analysis

### Eye movements Analysis

Recordings from the two EOG electrodes were processed using a custom-made script in Matlab to detect horizontal eye movements and evaluate performance in the behavioral identification task. EOG signals were low-pass filtered using as a cutoff frequency of 20 Hz. Subsequently, the recordings were segmented to obtain a time window of 3 seconds starting at the presentation of the question on the screen. Eye movements produce a moving dipole source generating positive and negative peaks when differentiating the two EOG electrodes, representing eye movements toward the right and left respectively. The first peak was individuated for each trial if the amplitude was exceeding 1.5 standard deviations from the average and its timing was at least 150 ms after the question. When no clear peak could be detected, the trial was discarded from subsequent analysis.

### Reaction Times analyses and results

Reaction times (RTs) were analyzed by means of a repeated measures ANOVA using a 2 (place of articulation of the auditory syllable: bilabial/dental) × 2 (lip stimulation: present/absent) × 2 (question type: positive/negative response) full-factorial design using R statistical package^73^. The analysis was performed after removing incorrect or invalid trials (13% of trials). The analysis of variance on RTs revealed a significant main effect of question type (F(1, 11) = 8.17, p = 0.016, positive = 507 ± 23 ms; negative = 556 ± 29 ms), due to faster response when the question required a “yes” response. A main effect of stimulation was also significant (F(1, 11) = 5.92, p = 0.034), due to faster reaction times when the electrical stimulation of the lips was present (mean = 520 ± 27 ms) with respect to absent (mean = 542 ± 28 ms), probably due to a startling effect of the stimulation. No other effects or interactions reached significance threshold (alpha = 0.05). RTs did not correlate with any of the ERSP measures reported in the result section. In the present experiment we introduced a delay (1.5 seconds) between the syllable presentation and the identification question to control for possible confounds related to ocular movements on the sensorimotor rhythms. Nevertheless, it is possible that such correlation could be detected under high-speed requirements (e.g., by decreasing the delay between sound processing and question presentation).

### EEG Time-Frequency analysis

To avoid edge effects, a pre-stimulus baseline was identified in a time windows from −1000 ms to −150 ms with respect to the critical event (vowel onset and electrical stimulation delivery). Subsequently, the ratio of logarithmic power changes was computed between the post-stimulus time window and the pre-stimulus baseline. This measure is defined as event related spectral perturbation (ERSP). ERSPs are changes over a broad spectral range scaled in normalized decibel units (averaged logarithmic power spectrum changes with respect to the pre-stimulus baseline interval). Significant perturbations compared with the baseline interval were estimated using the bootstrap resampling method with 10000 random permutations. In this study, ERSPs were computed using a Morlet sinusoidal wavelet set at 3 cycles at 4 Hz rising linearly to 20 cycles at 40 Hz with a frequency resolution of 0.5 Hz. The time window on which ERSPs were calculated ranged from −1000 to 1000 milliseconds. Wavelet estimates across trials for each time and frequency were then converted to a time-frequency matrix.

Mean ERSP averaged across subjects was calculated in two regions of interest (ROIs) by averaging left central electrodes (C1, C3 and C5) and right ones (C2, C4 and C6) respectively, to evaluate left and right hemispheric differences in the ERSPs modulations. In the first exploratory analysis, we compared the ERSP related to the two syllables and the effect of electrical stimulation separately in the two ROIs. To test the significance of these comparisons, we computed paired t-tests with a bootstrapped random distribution on 10000 permutations. This random distribution represents the null hypothesis that no differences exist between conditions. To control for the inflation of Type I error rates, associated with multiple comparisons across time and frequency points, a correction for false discovery rate (FDR, obtained by ranking p-values and obtaining a critical value based on rank number and the total number of tests performed) was applied^74^ allowing for a conservative test of condition effects.

Based on the initial exploratory analysis, narrower frequency bands capturing the critical ERD/ERS events is identified for both mu and beta rhythms. ERSPs are averaged within the frequency band, for each participant and each combination of conditions separately and by considering two time windows, focused on the ERD and on the ERS events. The ERSPs for the two frequency bands are analyzed separately by means of a repeated measures ANOVA with a 2 (syllable: bilabilal/dental) × 2 (lip stimulation: present/absent) × 2 (time window: ERD/ERS) full-factorial design. In order to test our hypothesis of an interaction between the place of articulation of the heard sounds with the stimulation to the lower lip, whenever an interaction between the syllable factor and the stimulation factor was present, post-hoc comparisons are calculated by using paired t-tests with Bonferroni’s correction for multiple comparisons. All analyses were performed using R statistical package^73^.

## Additional Information

**How to cite this article**: Bartoli, E. *et al*. Beta rhythm modulation by speech sounds: somatotopic mapping in somatosensory cortex. *Sci. Rep*. **6**, 31182; doi: 10.1038/srep31182 (2016).

## References

[b1] Kawato M. Internal models for motor control and trajectory planning. Curr Opin Neurobiol 9, 718–727 (1999). 10607637 10.1016/s0959-4388(99)00028-8

[b2] Grimme B., Fuchs S., Perrier P. & Schöner G. Limb versus speech motor control: a conceptual review. Motor Control 15, 5–33 (2011). 21339512 10.1123/mcj.15.1.5

[b3] Browman C. P. & Goldstein L. Articulatory gestures as phonological units. Phonology 6, 201 (1989).

[b4] Tourville J. a., Reilly K. J. & Guenther F. H. Neural mechanisms underlying auditory feedback control of speech. Neuroimage 39, 1429–1443 (2008). 18035557 10.1016/j.neuroimage.2007.09.054 PMC3658624

[b5] Perkell J. S. Movement goals and feedback and feedforward control mechanisms in speech production. J. Neurolinguistics 25, 382–407 (2012). 22661828 10.1016/j.jneuroling.2010.02.011 PMC3361736

[b6] Greenlee J. D. W. . Sensory-Motor Interactions for Vocal Pitch Monitoring in Non-Primary Human Auditory Cortex. PLoS One 8 (2013). 10.1371/journal.pone.0060783 PMC3620048 23577157

[b7] Behroozmand R., Karvelis L., Liu H. & Larson C. R. Vocalization-Induced Enhancement of the Auditory Cortex. Clin. Neurophysiol. 120, 1303–1312 (2009). 19520602 10.1016/j.clinph.2009.04.022 PMC2710429

[b8] Behroozmand R. . Neural Correlates of Vocal Production and Motor Control in Human Heschl’s Gyrus. J. Neurosci. 36, 2302–2315 (2016). 26888939 10.1523/JNEUROSCI.3305-14.2016 PMC4756159

[b9] Chang E. F., Niziolek C. a., Knight R. T., Nagarajan S. S. & Houde J. F. Human cortical sensorimotor network underlying feedback control of vocal pitch. Proc. Natl. Acad. Sci. USA 110, 2653–2658 (2013). 23345447 10.1073/pnas.1216827110 PMC3574939

[b10] Niziolek C. A., Nagarajan S. S. & Houde J. F. What does motor efference copy represent? Evidence from speech production. J. Neurosci. 33, 16110–16116 (2013). 24107944 10.1523/JNEUROSCI.2137-13.2013 PMC3792453

[b11] Behroozmand R. . Sensory-motor networks involved in speech production and motor control: An fMRI study. Neuroimage 109, 418–428 (2015). 25623499 10.1016/j.neuroimage.2015.01.040 PMC4339397

[b12] Behroozmand R., Ibrahim N., Korzyukov O., Robin D. A. & Larson C. R. Functional role of delta and theta band oscillations for auditory feedback processing during vocal pitch motor control. Front. Neurosci. 9, 1–13 (2015). 25873858 10.3389/fnins.2015.00109 PMC4379876

[b13] Sengupta R. & Nasir S. M. The Predictive Roles of Neural Oscillations in Speech Motor Adaptability. J. Neurophysiol. 60208, jn.00043.2016 (2016). 10.1152/jn.00043.2016 PMC4922470 26936976

[b14] Sengupta R. & Nasir S. M. Redistribution of neural phase coherence reflects establishment of feedforward map in speech motor adaptation. J. Neurophysiol. 113, 2471–2479 (2015). 25632078 10.1152/jn.00731.2014 PMC4416577

[b15] Wolpert D. M. & Kawato M. Multiple paired forward and inverse models for motor control. Neural Netw. 11, 1317–1329 (1998). 12662752 10.1016/s0893-6080(98)00066-5

[b16] Oztop E., Kawato M. & Arbib M. a. Mirror neurons: functions, mechanisms and models. Neurosci. Lett. 540, 43–55 (2013). 23063951 10.1016/j.neulet.2012.10.005

[b17] Yuen I., Davis M. H., Brysbaert M. & Rastle K. Activation of articulatory information in speech perception. Proc. Natl. Acad. Sci. USA 107, 592–597 (2010). 20080724 10.1073/pnas.0904774107 PMC2818927

[b18] Fadiga L., Craighero L., Buccino G. & Rizzolatti G. Speech listening specifically modulates the excitability of tongue muscles: a TMS study. Eur. J. Neurosci. 15, 399–402 (2002). 11849307 10.1046/j.0953-816x.2001.01874.x

[b19] Watkins K. Seeing and hearing speech excites the motor system involved in speech production. Neuropsychologia 41, 989–994 (2003). 12667534 10.1016/s0028-3932(02)00316-0

[b20] Roy A. C., Craighero L., Fabbri-Destro M. & Fadiga L. Phonological and lexical motor facilitation during speech listening: a transcranial magnetic stimulation study. J. Physiol. Paris 102, 101–105 (2008). 18440210 10.1016/j.jphysparis.2008.03.006

[b21] D’Ausilio A., Jarmolowska J., Busan P., Bufalari I. & Craighero L. Tongue corticospinal modulation during attended verbal stimuli: priming and coarticulation effects. Neuropsychologia 49, 3670–3676 (2011). 21958646 10.1016/j.neuropsychologia.2011.09.022

[b22] D’Ausilio A. . Listening to speech recruits specific tongue motor synergies as revealed by transcranial magnetic stimulation and tissue-Doppler ultrasound imaging. Philos. Trans. R. Soc. Lond. B. Biol. Sci. 369, 20130418 (2014). 24778384 10.1098/rstb.2013.0418 PMC4006190

[b23] Murakami T., Restle J. & Ziemann U. Effective connectivity hierarchically links temporoparietal and frontal areas of the auditory dorsal stream with the motor cortex lip area during speech perception. Brain Lang. 122, 135–141 (2012). 22030113 10.1016/j.bandl.2011.09.005

[b24] Nuttall H. E., Kennedy-Higgins D., Hogan J., Devlin J. T. & Adank P. The effect of speech distortion on the excitability of articulatory motor cortex. Neuroimage 128, 218–226 (2015). 26732405 10.1016/j.neuroimage.2015.12.038

[b25] Ito T. & Ostry D. J. Speech sounds alter facial skin sensation. J. Neurophysiol. 107, 442–447 (2012). 22013241 10.1152/jn.00029.2011 PMC3349680

[b26] Ito T., Tiede M. & Ostry D. J. Somatosensory function in speech perception. Proc. Natl. Acad. Sci. USA 106, 1245–1248 (2009). 19164569 10.1073/pnas.0810063106 PMC2633542

[b27] Gick B. & Derrick D. Aero-tactile integration in speech perception. Nature 462, 502–504 (2009). 19940925 10.1038/nature08572 PMC3662541

[b28] Möttönen R., Järveläinen J., Sams M. & Hari R. Viewing speech modulates activity in the left SI mouth cortex. Neuroimage 24, 731–737 (2005). 15652308 10.1016/j.neuroimage.2004.10.011

[b29] Ito T., Johns A. R. & Ostry D. J. Left lateralized enhancement of orofacial somatosensory processing due to speech sounds. J. Speech. Lang. Hear. Res. 56, S1875–S1881 (2013). 24687443 10.1044/1092-4388(2013/12-0226) PMC4228692

[b30] Ito T., Gracco V. L. & Ostry D. J. Temporal factors affecting somatosensory-auditory interactions in speech processing. Front. Psychol. 5, 1–10 (2014). 25452733 10.3389/fpsyg.2014.01198 PMC4233986

[b31] Pfurtscheller G. & Lopes da Silva F. H. Event-related EEG/MEG synchronization and desynchronization: basic principles. Clin. Neurophysiol. 110, 1842–1857 (1999). 10576479 10.1016/s1388-2457(99)00141-8

[b32] Hari R., Salmelin R., Mäkelä J. P., Salenius S. & Helle M. Magnetoencephalographic cortical rhythms. In International Journal of Psychophysiology 26, 51–62 (1997). 10.1016/s0167-8760(97)00755-1 9202994

[b33] Andrew C. & Pfurtscheller G. On the existence of different alpha band rhythms in the hand area of man. Neurosci. Lett. 222, 103–106 (1997). 9111739 10.1016/s0304-3940(97)13358-4

[b34] Müller G. R. . Event-related beta EEG changes during wrist movements induced by functional electrical stimulation of forearm muscles in man. Neurosci. Lett. 340, 143–147 (2003). 12668257 10.1016/s0304-3940(03)00019-3

[b35] Pfurtscheller G., Neuper C., Brunner C. & da Silva F. L. Beta rebound after different types of motor imagery in man. Neurosci. Lett. 378, 156–159 (2005). 15781150 10.1016/j.neulet.2004.12.034

[b36] Neuper C. & Pfurtscheller G. Evidence for distinct beta resonance frequencies in human EEG related to specific sensorimotor cortical areas. Clin. Neurophysiol. 112, 2084–2097 (2001). 11682347 10.1016/s1388-2457(01)00661-7

[b37] Leocani L. Event-related coherence and event-related desynchronization/synchronization in the 10 Hz and 20 Hz EEG during self-paced movements. Electroencephalogr. Clin. Neurophysiol. Potentials Sect. 104, 199–206 (1997). 10.1016/s0168-5597(96)96051-7 9186234

[b38] Pineda J. A. The functional significance of mu rhythms: translating ‘seeing’ and ‘hearing’ into ‘doing’. Brain Res. Brain Res. Rev. 50, 57–68 (2005). 15925412 10.1016/j.brainresrev.2005.04.005

[b39] Neuper C. & Pfurtscheller G. Event-related dynamics of cortical rhythms: Frequency-specific features and functional correlates. Int. J. Psychophysiol. 43, 41–58 (2001). 11742684 10.1016/s0167-8760(01)00178-7

[b40] Koelewijn T., van Schie H. T., Bekkering H., Oostenveld R. & Jensen O. Motor-cortical beta oscillations are modulated by correctness of observed action. Neuroimage 40, 767–775 (2008). 18234516 10.1016/j.neuroimage.2007.12.018

[b41] Avanzini P. . The dynamics of sensorimotor cortical oscillations during the observation of hand movements: An EEG study. PLoS One 7, 1–10 (2012). 10.1371/journal.pone.0037534 PMC3356327 22624046

[b42] Hari R. . Activation of human primary motor cortex during action observation: a neuromagnetic study. Proc Natl Acad Sci USA 95, 15061–15065 (1998). 9844015 10.1073/pnas.95.25.15061 PMC24575

[b43] Rossi S. . Somatosensory processing during movement observation in humans. Clin. Neurophysiol. 113, 16–24 (2002). 11801420 10.1016/s1388-2457(01)00725-8

[b44] Muthukumaraswamy S. D. & Johnson B. W. Primary motor cortex activation during action observation revealed by wavelet analysis of the EEG. Clin. Neurophysiol. 115, 1760–1766 (2004). 15261854 10.1016/j.clinph.2004.03.004

[b45] D’Ausilio A., Bartoli E. & Maffongelli L. Grasping synergies: A motor-control approach to the mirror neuron mechanism. Phys. Life Rev. 12, 91–103 (2015). 25465480 10.1016/j.plrev.2014.11.002

[b46] Bowers A., Saltuklaroglu T., Harkrider A. & Cuellar M. Suppression of the μ rhythm during speech and non-speech discrimination revealed by independent component analysis: implications for sensorimotor integration in speech processing. PLoS One 8, e72024 (2013). 23991030 10.1371/journal.pone.0072024 PMC3750026

[b47] Jenson D. . Temporal dynamics of sensorimotor integration in speech perception and production: Independent component analysis of EEG data. Front. Psychol. 5 (2014). 10.3389/fpsyg.2014.00656 PMC4091311 25071633

[b48] Chen R., Corwell B. & Hallett M. Modulation of motor cortex excitability by median nerve and digit stimulation. Exp. Brain Res. 129, 77–86 (1999). 10550505 10.1007/s002210050938

[b49] Engel A. K. & Fries P. Beta-band oscillations-signalling the status quo? Curr. Opin. Neurobiol. 20, 156–165 (2010). 20359884 10.1016/j.conb.2010.02.015

[b50] Arnal L. H., Wyart V. & Giraud A.-L. Transitions in neural oscillations reflect prediction errors generated in audiovisual speech. Nat. Neurosci. 14, 797–801 (2011). 21552273 10.1038/nn.2810

[b51] Della Penna S. . Temporal dynamics of alpha and beta rhythms in human SI and SII after galvanic median nerve stimulation. A MEG study. Neuroimage 22, 1438–1446 (2004). 15275901 10.1016/j.neuroimage.2004.03.045

[b52] Houdayer E., Labyt E., Cassim F., Bourriez J. L. & Derambure P. Relationship between event-related beta synchronization and afferent inputs: Analysis of finger movement and peripheral nerve stimulations. Clin. Neurophysiol. 117, 628–636 (2006). 16427358 10.1016/j.clinph.2005.12.001

[b53] Tian X. & Poeppel D. Dynamics of Self-monitoring and Error Detection in Speech Production: Evidence from Mental Imagery and MEG. J. Cogn. Neurosci. 1–10 (2013). 25061925 10.1162/jocn_a_00692 PMC4465382

[b54] Tian X. & Poeppel D. The effect of imagination on stimulation: the functional specificity of efference copies in speech processing. J. Cogn. Neurosci. 25, 1020–1036 (2013). 23469885 10.1162/jocn_a_00381

[b55] Tian X. & Poeppel D. Mental imagery of speech: linking motor and perceptual systems through internal simulation and estimation. Front. Hum. Neurosci. 6, 314 (2012). 23226121 10.3389/fnhum.2012.00314 PMC3508402

[b56] Salmelin R., Hämäläinen M., Kajola M. & Hari R. Functional segregation of movement-related rhythmic activity in the human brain. NeuroImage 2, 237–243 (1995). 9343608 10.1006/nimg.1995.1031

[b57] Nasir S. M. & Ostry D. J. Somatosensory Precision in Speech Production. Curr. Biol. 16, 1918–1923 (2006). 17027488 10.1016/j.cub.2006.07.069

[b58] Tremblay S., Shiller D. M. & Ostry D. J. Somatosensory basis of speech production. Nature 423, 866–869 (2003). 12815431 10.1038/nature01710

[b59] Fowler C. a. & Dekle D. J. Listening with eye and hand: cross-modal contributions to speech perception. J. Exp. Psychol. Hum. Percept. Perform. 17, 816–828 (1991). 1834793 10.1037//0096-1523.17.3.816

[b60] Treille A., Cordeboeuf C., Vilain C. & Sato M. Haptic and visual information speed up the neural processing of auditory speech in live dyadic interactions. Neuropsychologia 57, 71–77 (2014). 24530236 10.1016/j.neuropsychologia.2014.02.004

[b61] Bruderer A. G., Danielson D. K., Kandhadai P. & Werker J. F. Sensorimotor influences on speech perception in infancy. Proc. Natl. Acad. Sci. 1–6 doi: 10.1073/pnas.1508631112 (2015). PMC4640749 26460030

[b62] Kuhl P. K., Ramirez R. R., Bosseler a., Lin J.-F. L. & Imada T. Infants’ brain responses to speech suggest Analysis by Synthesis. Proc. Natl. Acad. Sci. 111, 1410963111– (2014). 10.1073/pnas.1410963111 PMC4128155 25024207

[b63] Werker J. F. & Tees R. C. Cross-language speech perception: Evidence for perceptual reorganization during the first year of life. Infant Behav. Dev. 25, 121–133 (2002).

[b64] Schwartz J. L., Basirat A., Ménard L. & Sato M. The Perception-for-Action-Control Theory (PACT): A perceptuo-motor theory of speech perception. J. Neurolinguistics 25, 336–354 (2012).

[b65] Sohoglu E., Peelle J. E., Carlyon R. P. & Davis M. H. Predictive top-down integration of prior knowledge during speech perception. J. Neurosci. 32, 8443–8453 (2012). 22723684 10.1523/JNEUROSCI.5069-11.2012 PMC6620994

[b66] Callan A. M., Callan D. E., Tajima K. & Akahane-Yamada R. Neural processes involved with perception of non-native durational contrasts. Neuroreport 17, 1353–1357 (2006). 16951584 10.1097/01.wnr.0000224774.66904.29

[b67] Burton M. W. & Small S. L. Functional neuroanatomy of segmenting speech and nonspeech. Cortex 42, 644–651 (2006). 16881272 10.1016/s0010-9452(08)70400-3

[b68] D’Ausilio A. . The motor somatotopy of speech perception. Curr. Biol. 19, 381–385 (2009). 19217297 10.1016/j.cub.2009.01.017

[b69] D’Ausilio A., Bufalari I., Salmas P. & Fadiga L. The role of the motor system in discriminating normal and degraded speech sounds. Cortex. 48, 882–887 (2012). 21676385 10.1016/j.cortex.2011.05.017

[b70] Bartoli E. . Listener-speaker perceived distance predicts the degree of motor contribution to speech perception. Cereb. cortex 25, 281–288 (2015). 24046079 10.1093/cercor/bht257

[b71] Laver J. Principles of phonetics. Cambridge Textb. Linguist. xxviii, 707 p (1994).

[b72] Delorme A. & Makeig S. EEGLAB: an open source toolbox for analysis of single-trial EEG dynamics including independent component analysis. J. Neurosci. Methods 134, 9–21 (2004). 15102499 10.1016/j.jneumeth.2003.10.009

[b73] R Development Core Team, R. R: A Language and Environment for Statistical Computing. R Foundation for Statistical Computing **1** (2011).

[b74] Benjamini Y. & Yekutieli D. The control of the false discovery rate in multiple testing under dependency. Ann. Stat. 29, 1165–1188 (2001).

[b75] Dale A. M., Fischl B. & Sereno M. I. Cortical surface-based analysis. I. Segmentation and surface reconstruction. Neuroimage 9, 179–194 (1999). 9931268 10.1006/nimg.1998.0395

